# Principes généraux de Physiologie-Pathologique, coordonnés d'après la Doctrine de M. Broussais

**Published:** 1821-03

**Authors:** 


### CRITICAL ANALYSES

OF

### RECENT PUBLICATIONS, IN THE DIFFERENT BRANCHES OF MEDICINE AND SURGERY,

### In the Literature of Foreign Nations.

---

Πατρίδες ἄρα
Ἀνδράσιν, οὐ πατραῖς ἄνδρες, ἀγαλλόμεθα.

---

*Principes généraux de Physiologie-Pathologique, coordonnés d'après la Doctrine de M. Broussais.* Par L. J. Bégin, Chirurgien Aide-Major a l'Hôpital Militaire d'Instruction de Metz. 8vo. pp. 390. Mequignon-Marvis, à Paris, 1821.

ABOUT a year since, we noticed a book professing to be the substance of the Lectures of Dr. Broussais, and of which we promised to present a particular account to the readers of this Journal; but, on further consideration, we determined not to do the Professor at the Val-de-Grace so much injustice as to promulgate such vague, imperfect, and desultory statements, under the title assumed for them. Dr. Broussais has himself published only a Treatise on Chronic Inflammation, a work entitled an "Examination of the Medical Doctrine generally adopted," and some Memoirs in the *Journal Universel des Sciences Medicales*, and other Journals; of the whole of which an abstract has been given in the Exposition of his Doctrine inserted in some late Numbers of this Journal, and the Historical Sketches of the Progress of Medicine: so that, on perusing the work of Mr. Bégin, and finding him say, in the Introduction, if there is any thing good in it, it must be attributed to Dr. Broussais, we are obliged to infer that the Professor has developed many views in his Lectures that are not to be found in his printed publications. Yet, even admitting this supposition, we cannot but admire the modesty of Mr. Bégin, in claiming for himself so little merit as he has done on this occasion. The work is composed in so methodic and perspicuous a manner, and presents such a well-arranged exposition of the principles in physiology that are of most essential importance in regard to pathology, that, as a mere compilation, it might obtain no small degree of credit to a writer who had even acquired so much estimation as had already been attained by Mr. Bégin. This work is not, indeed, a mere exposition of the doctrines of Broussais; for the author has advanced objectionable criticism to his opinions, and has in some instances deviated from his principles in the theories he endeavours to establish; and he has himself shown, in a very favourable point of view, the benefits that will arise to pathology from the further application of the knowledge of the human economy in the state of health than had hitherto been effected.

Amongst the reflections contained in the "Introduction," (which is full of judicious considerations,) the author adduces some good remarks on the utility, and indeed absolute necessity, of theory, which he concludes with saying, that, "notwithstanding their continual declamations against theories, the empirics always suffer themselves to be guided by the ideas they form of the nature of the disease; and they constantly appropriate, in spite of themselves, their remedial measures to that supposed nature." An English author (whose writings we have so often quoted of late, when some of the most interesting and difficult questions relating to medicine have come before us,) had previously remarked, in his "View of the Relations of the Nervous System," that general conclusions are made "by none more frequently than by those who profess to hold theory in contempt, but who, in fact, practise more upon theory than any others, and in general upon that of the very worst kind,—namely, that founded upon the loosest analogies; upon that made with no care, with no trouble; upon that which they would be offended to hear called theory: unaware of the processes of their own minds, they fancy that inferences are visible things; they are perpetually meeting with solitary facts, and as often generalizing them, until they meet with opposite facts; and it is highly probable that they would generalize them too; or else they are at their *ne plus ultra,* and conclude it is all a dark business."

The first chapter in the work before us relates to "the Vital Properties." The author here exposes the errors of conduct of those who have regarded the results of the properties of organization as elementary existences independent of such organization, and who have talked of changes of the vital properties as if it were possible that alteration could take place in them without any change of the structure on which their existence depends. But his own notions on this subject are not free from much obscurity, and even positive contradictions. He says, "*For the physiologist who embraces at one view the whole series of organized beings, there exists no other vital properties than those which are inherent with all bodies endowed with life, at all the epochs of their existence, and which are absolutely indispensable for their preservation. In proportion as the organization becomes complicated, its acts become more numerous; but it is not because those properties are multiplied,—it is because the organs are more varied and execute more difficult functions.*" If the vital properties are regarded as the causes of organization, there must be a diversity in them consonant with the diversities of the organization produced, or effects must take place without causes. If they are considered as the effects of organization, we only shift the difficulty, as will be presently shown. It might be supposed that Mr. Bégin may regard the vital properties as the properties of a principle that is attached to organized bodies, on which they act as with machines variously constructed; but that such are not his notions, will appear by his definition of irritability. He says, "*Thus defined, the vital properties are reduced to one single power, or rather to one single fact, which I call* IRRITABILITY, *after Glisson and Gorter.*" (Here he converts his obscure inferences into a FACT.) Irritability he defines, "AN APTITUDE POSSESSED BY CERTAIN BODIES TO RECEIVE THE IMPRESSION

OF OTHER BODIES WHICH ARE FOREIGN TO THEM, AND TO MOVE THEMSELVES IN CONSEQUENCE OF THIS IMPRESSION." —"*Irritability*," he then immediately adds, "*is a quality, a condition, an inherent property, of all matter endowed with life: it depends on the organization of that matter; it arises, is developed, and disappears, with the organic texture: but its nature is as inconceivable as gravity, extent, and all the other physical properties of bodies.*"

According to this reasoning, it appears, then, that all the vital properties are reduced to irritability; are but irritability; that irritability is dependent on organization, and that it is identical, though the organization is various, and executes various functions. The only means, then, of accounting for this diversity of functions are, either diversities of the *mechanism* of the part, (the validity of which proposition Mr. Bégin does not admit,) or the existence of *some other properties.* These other properties are considered by Mr. Bégin as the results of the laws of the *living chemistry (chimie vivante)* of Broussais: and thus he satisfies himself with mere *words*, instead of ideas or precise inferences. As—and the proposition is admitted—the laws of this *living chemistry* are different from those of the chemistry of inorganic bodies, that in which the difference consists, and which is peculiar to life, must be either a peculiar entity which is a cause of life, and, being a cause, must therefore enter into life as one of its constituents, and consequently must become a property of life;—or it must be the effect of life, and, being an *effect proper to life*, it must have its proper or peculiar causes; which peculiar causes (in spite of an abuse of terms,) must be properties of life, or *vital properties.* Thus, by this mode of reasoning, a diversity of vital properties is demonstrated.

It is true that Mr. Bégin assumes a distinction between *functions* and the mere development of vitality, and he simplifies the idea of life so far as to make it consist in "an aptitude possessed by certain bodies to receive the impressions of other bodies which are foreign to them, and to move themselves in consequence of this impression." But he cannot, by this assumption, get out of the dilemma in which we have shown him to be placed. This simplification of life is an untenable assumption: it is a gross absurdity. How can an *aptitude to move on the receipt of certain impressions,* combined with any mechanism, account for any one *proper vital act?* Besides this, his distinction of *functions* and the *simple act of vitality*, supposes effects to exist of which there are not the causes. For, as the diversities of functions are not explicable either by diversities of *mechanism,* or by diversities of *vital properties*, and (as it has been above shown) his *living chemistry* being referable to *vital properties*, there remains nothing that can be the causes of those diversities of functions: therefore, according to his own reasonings, *they must originate from nothing.*

That Mr. Bégin should have got into such a dilemma, by attempting to reduce the vital properties to *one single principle*, does not much surprise us. These, however, are but uninteresting topics to most readers; and we should not have dwelt so long on this part of the work, had it not been the production of such a physiologist as one of the writers of the article "Irritabilité," in the *Dictionnaire des Sciences Medicales.*

The second chapter of the work before us, is on "the Varieties of the Animal Organization;" a title somewhat too comprehensive, as the disquisition relates only to what have ordinarily been termed diversities of *temperament*, and *idiosyncracies.* Mr. Bégin employs the latter term in a new sense, and he makes a distinction between temperaments and idiosyncrasies, that he has, perhaps, derived from Bordeu. He proposes, besides marking the temperaments of individuals,—which should be characterized by the relative prevalence of the *sanguineous*, *nervous*, or *lymphatic* systems, throughout the body generally; that we should designate the organs or functions which predominate in the economy, and regard such a predominance as designative of the *idiosyncracies.* Thus, a person may be said to have a sanguineous temperament and pulmonary idiosyncracy; or a nervous temperament and an hepatic idiosyncrasy; or a lymphatic temperament and a mucous (from a predominance of activity or irritability of the mucous membranes) idiosyncrasy; or one of the above-mentioned temperaments with fibro-articular idiosyncrasy, (in which the ligaments of the joints are especially disposed to disease; and which idiosyncrasy constitutes, according to Mr. Bégin, what has been called the *gouty diathesis*:) and so with the other combinations of the *three* (above-enumerated) general, and the more numerous particular, varieties of the organization in individuals. There seems to be nothing objectionable in this proposition, whilst it is qualified to favour conciseness in the narration of particular histories of disease.

The importance of the influence of peculiarities of temperament on particular cases of disease, has not, perhaps, been sufficiently considered of late, by the generality of English practitioners. Darwin probably carried this doctrine too far, as well as his sympathetic theory; and hence, very likely, the improper discredit into which they have both fallen: for the abuse of principles, however good, by excess in their application, very commonly leads to an unmerited degree of neglect of them. The fate of Darwin and his medical writings has, however, been somewhat extraordinary. He obtained an overwhelming reputation during his life,* and he is already almost forgotten; and his works have contributed much to the unjust odium of medical theory that is now prevalent, whilst they present many of the most admirable examples of its utility.

Mr. Bégin says, "the doctrine of the temperaments is one of the most important parts of medical theory. It is the foundation of the most valuable indications for medical practice. Men differ so much according to the diversities of their constitutions, that the otherwise

* And this not merely with the public, but with the profession also; many proofs of which might be adduced. As one instance, we may mention that the physician who, next to Darwin, had obtained the greatest celebrity in the West of England, carried his daughter to Derby to place her under Darwin's care, with a confident expectation that he could cure her, though she was in the last stage of tubercular consumption.

best-narrated histories of disease would be incomplete, and barren to our contemporaries and to posterity, if we neglected to designate the peculiar organization of the subjects of them, before we described the causes, the symptoms, and the termination, of the maladies to which they relate."

Amongst the temperaments and idiosyncracies, some are born with us,—at least, the predispositions to them are transmitted by generation; others are produced either by the revolutions which ordinarily take place at different epochs of life, or result from certain modes of living, climate, &c. To overthrow an unfavourable temperament, and correct a vicious idiosyncrasy, and replace them by more advantageous conditions, are the objects of physical education and medical gymnastics, rather than of the ordinary practice of medicine; and are, therefore, not considered by Mr. Bégin in this work. He directs his attention to the exposition of the consequences of the particular conditions above alluded to, when they actually exist, and endeavours to draw some practical indications from his views. The sanguineous temperament, he says, always accompanies the muscular idiosyncrasy, and it is commonly accompanied with gastric and genital idiosyncracies. Inflammation, in the subjects of it, but rarely passes into the chronic state, or affects secondarily the *white vessels*.* The heart is particularly liable to be affected by sympathy, and it is one of the conditions in which the most precise indications may be drawn from the state of the pulse; the most important of which are passed in review by Mr. Bégin. He dwells especially on the important fact, that a very high degree of excitement of the capillary vessels and pain, instead of increasing the action of the heart, "chain" the motion of that organ, which then becomes tumultuously agitated, and dilates with difficulty: in which state, blood-letting, abstinence from food, and cold drink, serve to develop its movements; a result which is frequently found to take place in a very sudden manner, after the reduction of a strangulated hernia. On discussing the question, "What is the cause of the fullness and largeness of the pulse observed in certain cases of irritation; whilst others render it small, hard, contracted, and almost convulsive?" he says, "when the inflamed parts are soft, spongy, easily dilatable, and not abundantly supplied with nerves, the pulse is full, developed, the artery pulsates forcibly, and raises the finger applied to it: such are the pulsations which characterize inflammation of the laminous tissue of the lungs, the liver, the spleen, and all the parenchymatous organs." The occasional deviations from this condition have not escaped his attention, and are afterwards considered. Broussais supposes that the larger congeries of capillary vessels, when irritated, establish an obstacle to the circulation which renders the pulse full and hard. This explanation is opposed by Mr. Bégin; because we find such a pulse when no particular obstacle to the circulation can be supposed to exist, as after vigorous exercise, and in one arm alone when this member is exclusively exerted; and because such

* See the exposition of the Docrine of Broussais in this Journal for January 1819, for an explanation of this expression.

a pulse is present with slight degrees of inflammation of the mucous membrane of the stomach and small intestines, when no considerable obstacle can be supposed to exist; whilst the pulse becomes small, hard, and contracted, when the inflammation has acquired a greater degree of extent and violence. Mr. Bégin thinks that the diversities of the character of the pulse should be attributed to relative diversities of sympathy with the organs affected, from which they arise; and this explanation is not at all inconsistent with the facts that a full soft pulse ordinarily accompanies inflammation of loose parenchymatous structures, and a small hard pulse that of firm extended membranes. In the former case, there is but little pain, and the capillary vessels are freely dilated; circumstances which sympathetically affect the heart, so as to excite and develop its movements. In the latter, there is severe and often intense pain; the free dilatation of the capillaries is prevented, and the heart and arteries are affected so that the dilatation and contraction of the former are impeded by the intensity of the suffering; and the latter are contracted, by a sort of erethism, on the column of blood which traverses them. Every part, Mr. Bégin further argues, has its peculiar sympathetic relations with the heart; and hence the connection of particular states of the pulse with diseases of particular organs, and even particular forms of structure. Hence, different kinds of pulse accompany inflammation of the mucous, muscular, and serous, tunics of the intestines.

On treating of the nervous temperament, the author digresses somewhat from his proper course, and advances some suppositions respecting the cause of the first inspiration of the infant, and the feelings which lead him to take food. He attributes the former to the uneasy sensation excited in the lungs by the presence of blood in them that has not undergone the modification it had received in the placenta; and he thinks that spirituous frictions on the skin only tend to establish the act by rousing the brain, and rendering it sensible to the call made on it by the lungs. He exercises his imagination in a similar manner, to account for the instincts and passions of animals. We are all acquainted with Darwin's attempts in this way, especially with his reasonings on the pleasure with which men regard the breast of a pretty young woman; to which, however, there happens to be this objection, that a man who had been *brought up by hand* has no such sensations on looking at a *spoon*.

Mr. Bégin discusses, also, in this section, the nature of consciousness and of delirium; and then treats of the influence of the nervous system on the rest of the body in the ordinary actions of life. In this part of the work there are several erroneous indications, or, at least, several assumptions which are not at all well established. He says, the tendons, ligaments, cartilages, and bones, are not penetrated by nerves;* and he adds, that "the free communication of the nervous

* This statement is so much in opposition to the most probable inferences from known facts,—as the occurrence of pain in ligaments, &c.—that we think it prudent to adduce Mr. Bégin's own words on this occasion: he says, "Les parties qui sont passives dans l'economie, et qui ne sont utile qu'à raison de la resistance de leur texture, ne sont pas pénétrées par les nervos; tels sont les tendons, les ligaments, les cartilages, les os."

extremities with the cerebral centre, is not indispensible to the nutrition of the organs in which these extremities are expanded. The *analogical proof* (this is Mr. Bégin's expression) of this fact, is furnished by animals entirely devoid of nerves, and by the tissues which in others want them, the nutrition of which is complete and very active. The direct demonstration of it, consists in the continuance of the phenomena of nutrition after all the nerves of a part have been destroyed, or after they have ceased to perform their functions, as is seen in some cases of paralysis of the limbs with total loss of sensation." Whether nervous influence is necessary for nutrition in animals supplied with nerves, is a matter of doubt; but we are surprised to find Mr. Bégin neglect some of the plainest points of anatomical demonstration, and talk of the continuance of nutrition in a part after the total destruction of its nerves. It is not possible wholly to destroy the nerves of a part, or even to interrupt their communication with their respective centres, without making a similar destruction of the arteries; for the ganglionic nerves are so intimately blended with the coats of the arteries, that it is impossible to divide them and leave the vessels continuous. The fact, too, from which Mr. Bégin infers a proof of nutrition without nervous influence, only warrants an inference that the nerves serving for sense and motion are not necessary for nutrition. The notion that this function is subservient to the ganglionic nerves, is not at all opposed by it.

Before a man attempts to frame a theory, he should be well acquainted with the grounds of human knowledge, or he may possess a high degree of intellectual power,—as a talent for acute and original observance, a great facility of comparison of ideas, and a very extensive range of abstract reflection,—and yet produce nothing that will bear the touchstone of truth, whilst he is himself unable to discern the difference between hypothesis and theory. We recommend Mr. Bégin to peruse a book which is, we have reason to believe, becoming pretty well known in Paris,—the "General Indications" of Mr. Pring; a work which is interesting to all who are engaged in the pursuit of natural science; to metaphysicians; and to moral philosophers; as it develops a system of Principles and Indications which cannot fail to promote the attainment of truth, and which must be particularly interesting to physicians, in consequence of the author having applied those Principles to the subjects of Physiology and Pathology in a particular manner, and hence determined, with a degree of precision that admits of no disputation, the extent of our real knowledge in those sciences; whilst his Indications—which are the production of a fertility of thought and originality of views as extraordinary as the solidity of the judgment by which his principles are established,—mark out new routes for the acquisition of knowledge that may occupy, for ages hence, the most active Spirits of our kind in their development.

Mr. Bégin seems to be unacquainted with some very important and well-known facts in physiology: after noticing the return of sensibility in a part which had been deprived of that faculty by a division of the nerve distributed to it, he says, "we cannot suppose that reunion of the divided nerve takes place." He uses but very little reserve in drawing such inferences as suit his purposes; for he says that, even supposing that a re-union of the nerve did take place, "a multitude of facts lead us to believe that the necessary communication could not be effected through the cicatrix."

The rest of this section comprises nothing proper to Mr. Bégin, nor any opinions of Broussais that have not been already noticed in this Journal.

A considerable part of the section on the *lymphatic temperament*, is occupied in arguing against the opinion that the lymphatic system is in a state of relative debility in individuals of this temperament. Mr. Bégin infers, from its being especially developed, that its vitality is particularly developed; and hence, in conformity with a generally-received axiom in physiology, that it is particularly liable to disease. Mr. Bégin remarks, that, in subjects of this kind, not only is the lymphatic system particularly developed, but that this is the case also with all the tissues supplied only with colourless fluids, as the cellular tissue, &c.; a thing which must readily be preconceived, if what is very probable be admitted, that the whole of the soft structures of the body are constituted merely of congeries of vessels containing either red or white fluids.

One of the most common and most remarkable of the coincidences, of a morbid kind, with the lymphatic temperament, is a want of due solidity, and even, in some instances, a softening of the bones. Mr. Bégin only ventures to give some hints respecting the etiology of this affection, by remarking the well-known fact, that, whenever a spot of ossification appears, there are vessels containing red blood running to that point; and he infers that red blood is necessary for the constitution of bone. But, as animals which are devoid of red blood have bones as firm as those which have such a fluid, (a fact which Mr. Bégin denies,) this inference is not very probable, though it does not want the support of several facts which seem to indicate, in a slight degree, such a notion: thus, ligaments and periosteum ossify on being kept in a state of inflammation for a certain length of time.

The same causes, the author observes, that excite intense inflammation in sanguineous subjects, and in those of the nervous temperament what are called nervous diseases, give rise to hardly sensible inflammation in persons of the lymphatic temperament, and the sympathetic phenomena of such inflammations are but feebly developed. Hence, in the first, the same external causes will produce a degree of inflammation of the mucous membrane of the small intestines that will give rise to fever; and in the last only a mucous diarrhœa, without fever. In these individuals, the lymphatic vessels have the greatest tendency to become the especial seat of irritation; and hence arise the lesions of structure described on a former occasion* in this Journal.

The next chapter is on *the idiosyncracies;* but nothing is adduced here but such obvious inferences from what has already been stated in this article, as render any detailed extracts unnecessary. There are,

* See *London Medical and Physical Journal,* January 1820.

however, some further remarks respecting the temperaments, that indicate a multitude of truths that may not be so readily discernible by every reader. Mr. Bégin says, "the temperaments above enumerated are remarkable in this, that any one portion of either of the three several systems, to the development of which they relate, cannot be irritated without all the parts of the same system being, for that reason, more disposed to contract that same irritation. An intimate sympathy unites with each other each of the vascular and nervous extremities of the body; and they cannot be much affected in one organ without being similarly affected in every other organ. Thus, when a sanguineous subject has inflammation in any part, the whole circulatory system is deranged, and the disposition to take on inflammation is increased in every organ. If several tumefactions take place in the lymphatic system in a person in whom this system is particularly developed, the lymphatic glands throughout the body will thence be particularly disposed to irritation; and what are called nervous diseases in any organ, in nervous subjects, increase the general susceptibility of the nerves, and they become affected with diseases similar to the one primarily established more readily than ordinarily. It may, then, be inferred, as a general law, that the appearance and renewal of inflammations, of *neuroses*, and of lymphatic tumors, communicate a new activity to the system especially affected in those diseases respectively, and augment the predominance of the sanguine, nervous, and lymphatic, temperaments. The history of morbid diatheses constantly furnishes proofs of the truth of this assertion; and it is in conformity with this law of the economy, that lesions which are primitively local become *repeated* in diverse parts of the body."

The third chapter treats on the *mucous membranes.* These structures constitute a very important series of organs in the pathology of Broussais; and, as it is through them and the skin that all extraneous matters must obtain admission into the system, there can be no hesitation in allowing them to perform a very essential part in the production of the greater proportion of diseases. They are, however, regarded in too exclusive a manner in the work before us; for, after noticing their offices, and giving some examples of their sympathetic relations, Mr. Bégin enters on the consideration of diseases in general, without treating of the laws which regulate the functions of the other structures of the body besides the sanguineous, nervous, and lymphatic systems, any further than in a very partial way, as the phenomena which they develop are brought forward in connection with those dependent on the properties of the mucous membranes. This, however, is a necessary consequence of the manner in which Mr. Bégin regards the structure under consideration. It is, he says, "the fundamental part of the living economy; all the others are in a manner modelled on it; their functions, and consequently their organic forms, vary according to the wants of which it is the source, and the more or less complete manner in which it prepares the nutritive substance." It is true that all the soft parts of the body are probably constituted of nothing more than developments of nerves, vessels, and the most simple animal fibre, which, perhaps, enters as a constituent into the whole: but, whether or not this be the case, it seems certain that each of what Bichat chose to regard as the elementary tissues of the body, as the cellular, serous, and fibrous, besides those already enumerated, has peculiar relations with other parts of the system in states of disease as well as in that of health; and the exposition of their laws should, consequently, form part of a General Treatise on Physiological Pathology. The sympathies, too, are not treated on in so complete a manner as we might have expected: some of those of the mucous membranes are stated in this chapter, and others are noticed, in a casual way, in subsequent parts of the work. It would, we think, have been better to have adduced what is known respecting their laws in one distinct section; and thus the particular illustrations of them brought forward in the consideration of particular diseases, would have been more perspicuous. Barthez, the late Professor of Medicine at Montpellier, took a very lucid view of this subject; and the inferences he drew from it present many very important indications for the treatment of disease. We shall deviate a little more than usual from our analytical course, in order to give an exposition of the view of Barthez, which, we suspect, will be new, as well as interesting, to a considerable proportion of our readers.

As all the parts of the animal economy have a more or less intimate degree of vital relation with each other, it is necessary to mention, in the first instance, the sense in which the term sympathy is here employed: it is considered, then, that *a sympathy takes place between two organs when an affection of one sensibly and frequently occasions a correspondent affection of the other, without this succession being, with probability, imputable to chance, to the mechanism of the organs, or to their concurrence of action in a generic mode of function or affection of the living body.* This definition excludes from the class of sympathetic actions several which Whytt, and subsequent writers, have generally confounded with them, and which are termed, by Barthez, *synergies.* Thus, action of the abdominal muscles accompanies the contraction of the bladder in the expulsion of the urine; and this connection has usually been called sympathetic, but it is *synergetic.* There is a concurrence of action in the abdominal muscles and the bladder, having a common and especial object, which is a natural function of the body,—the expulsion of the urine. A similar connection of active power exists between the abdominal muscles and the large intestines; between the same muscles and the glottis, exerted in coughing, certain efforts, &c. A similar synergy *(co-operation)*, for the fulfilment of an especial function, sometimes exists in disease; and hence some morbid actions may be classed amongst the synergies. This distinction of synergies and sympathies, is not a mere scholastic prolusion: it is qualified to establish some important practical indications; for, as the secondary affections of the former class have a specific tendency to the fulfilment of some object, it is obvious that no attempts should be made to remove them when the accomplishment of such an object is desirable: whilst those of the latter class, being merely an extension of disease, and having no such specific tendency, may commonly be removed with advantage; though they sometimes are connected—and probably as *causes*—with a disappearance of the primary affection; and when this happens, and the secondary affection occurs in a part the health of which is of less importance to the welfare of the patient than that of the primary, it may not be advisable to attempt their removal, at least not without some precautionary measures; as the primary affection sometimes recurs on the disappearance of the secondary, and, apparently, as a related consequence of its disappearance.

The sympathies are arranged by Barthez in the following orders: 1°, *those of organs having no particular sensible or material connection;* 2°, *those of organs which resemble each other in their structure and functions;* 3°, *those of organs having particular connections*,—that is to say, connections formed between two or more organs by an intermediate texture, as vessels and nerves common to them; or of a membranous or muscular structure which unites and renders continuous several adjacent organs; and those of organs assembled together in one system, which differs in its structure or functions from the other parts of the body.

The most remarkable of the sympathies of the first order, is that of the organs of voice, the neck, and the thyroid gland, with the genitals. The change which takes place in the *timbre* of the voice at the epoch of puberty, and which does not happen in eunuchs, is generally well known. The diapason of it commonly falls an octave at this period; and hence many who, when boys, were qualified to sing the *contr'alto* in concert, are calculated only for the *bass* after they have arrived at puberty. The neck of women, and especially the thyroid gland, enlarges much and rapidly at the same epoch, particularly after the indulgence of the venereal passion; and hence the observation in the verses of Catullus:

> Non illam nutrix orienti luce revisens,

> Hesterno collum poterit circumdare filo:

and the experiments commonly resorted to by the people in Italy, to ascertain whether or not, as they say, a girl is a virgin.

A similar sympathy is observed in some diseases, as the *mumps*, and perhaps in some ulcerative affections of the genitals.

Other sympathies of this order are often manifested in disease of the liver consequent on some injury of the head: pain concentrated in a spot in the head in some affections of the uterus; but the most frequent of this kind are those of the stomach with several remote parts, which were treated on and illustrated by Dr. RICHARD HARRISON, in one of his Gulstonian Lectures, inserted in a late Number of this Journal: next to these, are those of the intestines; instances of which are so common and well known, that the production of examples is unnecessary.

The sympathies of the second order are, also, very frequently witnessed; as in the general occurrence of inflammation in one eye when it has been established in the other: and RICHTER mentions having seen amaurosis of an eye, previously perfectly healthy, succeed to the same affection in the other, when it happened in this as a consequence of external injury. The sympathetic contraction and dilatation of the pupils in the healthy state, is generally known. Morgagni relates the case of a child, twelve years of age, who had suffered convulsions in several parts of the body, which, at length, became confined to one of the hands, the fingers of which were contracted. When an attempt was made to extend these fingers, the otherwise healthy hand was instantly affected with convulsions, and became violently contracted. If only a single finger of the diseased hand was extended, the corresponding finger of the other hand was affected with convulsions, which continued as long as the attempt at extension was continued. Theden (in his *Neue Bemerkungen und Erfahrungen,* u. w. s.) relates the following, somewhat analogous, story. A vesicatory was applied to the right arm of a woman that was paralytic. The plaster produced no apparent effect in the part to which it was applied, but it excited redness and severe pain in the corresponding part of the other arm, during the whole time it remained on the paralytic one. The paralysis, however, now disappeared from the right arm, and affected the left. A vesicatory was then applied to this extremity, the action of which was manifested on the right, causing redness and pain, as on the former occasion. On the paralysis being cured, vesicatories were not found to be attended with any such particular effects. The character and talents of Theden are such as to claim credit for this story.

Frank (a son of the Professor at Vienna) mentions having seen the case of a woman who had paralysis of one of the arms, and who determined to use efforts to move this arm at the same time that she moved the healthy one: she soon, apparently by this means, recovered the power of motion in it. Frank says that he has tried the same practice with other patients, and that it has sometimes been attended with success.

It is remarkable, that when one of the symmetrical members has attained, by habit, increased facility in the execution of certain movements, the corresponding member has acquired a similar facility in the execution of movements in the contrary direction, and not in the same direction. No plausible explanation has hitherto been given of this curious fact.

Other sympathies of this order are frequently manifested between parts which, without being symmetrically placed in the two lateral divisions of the body, have a similarity of structure and function. These are commonly witnessed in the skin, and in the fibrous and mucous membranes. Fanton mentions the case of a man having acute fever, in whom the portions of the skin on which there had recently been blisters, but which were quite healed, became again inflamed, and suppurated, when other and distant parts had blisters produced on them. The occurrence of inflammation of fibrous membranes in different parts of the body when it has been produced in one part, even by causes acting merely locally, as mechanical violence, is a very frequent phenomenon. Willis mentions having seen enlargement of the lymphatic glands in the neck occur, in consequence, he thought, of very great compression of the inguinal glands by a truss. Cases similar to this are related by Bartholin and Le Dran. The disordered state of the mucous membrane of the tongue which attends disease of that of the stomach, is another instance of sympathy of this kind.

When several successive alternations of affections have been established between two organs, naturally not particularly sympathizing, by any accidental causes, those organs contract a habit of correspondence of such affections.

The sympathies of the third order are very numerous: we shall merely give an example of each species of them, by way of illustration. The most remarkable instances of those dependent on particular nervous and vascular connection, occur in the abdominal and thoracic viscera, as between the liver and the stomach and small intestines; the stomach and the heart, &c.; which seem to be explicable by the common origin and close alliance of the nerves of those organs. Those from the continuity of membranes, in vomiting produced by irritation of the fauces; in the pain at the extremity of the urethra from stone in the bladder; itching of the nose from worms in the intestines; various affections of the skin from disorder of the mucous membranes. The progressive extension of diseases of the skin, as well as of mucous and serous membranes, seems often to be dependant on the same laws. There seems, however, to be a sympathetic connection between the skin and some internal parts, that is not, apparently, accountable for on this basis: thus, titillation about the ribs causes violent action of the diaphragm, which is not produced by titillation in other parts; and Barthez says, that an ointment in use in France, called *unguent d'arthanata*, (composed of violent purgatives,) produces vomiting when it is applied to the skin about the region of the stomach; purges when applied about the navel; and causes a great flow of urine when rubbed on the loins.

The sympathies of the whole of the digestive organs, and of the system of urinary organs, are examples of the species last enumerated. It did not, however, constitute any part of our intentions to enter into many details on this subject. We have adduced examples sufficient to show how the whole of the phenomena of this kind may be arranged in conformity with the views of Barthez, and we shall have occasion hereafter to notice many others of the most interesting kind to the medical practitioner, as we proceed in our analysis of the work of Mr. Bégin, who notices several which will, probably, be novel to many English readers, although this subject has been treated on by WHYTT, DARWIN, and Dr. ANDREW WILSON, in an extensive manner.

We should remark here, that the foregoing view of Barthez is not adduced as one that is free from obscurities. Some of the phenomena regarded as sympathetic, may be really synergetic, according to the definitions of those terms given above: the alterations that take place in the neck and voice at the epoch of puberty, may tend, with the development of the organs of generation, to the fulfilment of some common object; though we cannot discern what that object is, nor discover the train of relations to it. A person, by exercising his imagination, might conceive that the change to the grave and sonorous voice in man, is only in conformity with other of his distinctive characteristics from woman: but why there should be so much greater a development of the cellular texture about the neck than in other parts of the body in woman, is not so obvious. It is not enough to say that it contributes to what is called beauty, because what is morally beautiful is generally found to tend also to some relative physical good; and it is, probably, because certain objects tend to such good, that they are regarded as beautiful.

We return from this long digression to the work of Mr. Bégin, and resume our analysis of it where he treats of the sympathetic relations of the mucous membranes. Mr. Bégin makes no distinction of sympathies and synergies, and therefore comprises under the former appellation many phenomena which relate to the latter. We shall, if we produce in our course any observations of this kind, be content to use the terms employed by the author, and leave those readers to make the distinction above mentioned that think it of sufficient importance to engage their attention.

Mr. Bégin first notices the sympathetic relations of several parts of the mucous membranes and the brain, by which painful impressions originating from the mucous membrane of the fauces when there is want of drink, from that of the stomach when there is want of food, and from that of the lungs when there is want of air, are perceived by the sensorium, and by this medium determine the actions proper for their removal. What has been called spasmodic asthma, he says, arises from irritation of the mucous membrane of the lungs, which produces a spasmodic contraction of the glottis, and, by this means, the great difficulty of inspiration which attends that disease. The organic lesions of the lungs, heart, and pulmonary arteries, connected with it, and found after death, are, Mr. Bégin adds, consequences, not causes, of the asthma. This etiology, though it is but hypothetical, has much appearance of probability.

One of the most remarkable of the sympathetic effects of irritation of the mucous membranes of the stomach, œsophagus, and pharynx, is the production of the act of vomiting. Mr. Bégin argues that the membrane of the pharynx and œsophagus has the sympathetic relation with the abdominal muscles necessary for the production of the act of vomiting independent of the stomach, because the proper actions of those muscles are excited by irritating those portions of membrane in an animal whose stomach has been removed, and when the eighth pair of nerves has been divided on each side; which division, he remarks, is followed by paralysis of the stomach. Mr. Bégin thinks that the only sensible action the stomach is capable of, is a peristaltic motion similar to that of the intestines; and that the course of this action is reversed in vomiting, as well as in what may be termed *regurgitation:* (to distinguish it from vomiting properly, which is attended with action of the abdominal muscles, which does not accompany *regurgitation*, as we may readily observe in children, in whom this act is very common.) What has been termed *elective vomiting*, (in which substances not fit for digestion are thrown from the stomach, whilst such as are adapted for that process are retained,) seems to be only a sort of regurgitation; the stomach being excited to anti-peristaltic action only just in those parts where some improper and irritating substances are present.

The sympathy of the mucous membrane of the stomach and intestines with the skin, is next noticed by Mr. Bégin. "At the instant," he says, "when irritation is developed, and when congestion is established, in the mucous membrane of the parts above mentioned, the cutaneous surface becomes cold, a general shivering affects the patient, the vital actions seem hardly to extend to the external parts of the body: such is the onset of the greater part of idiopathic fevers, which are only *phlogoses* of those membranes. But this state of debility and torpor is soon dissipated: the irritated organ re-acts on the external parts; the vital actions are then roused in them, and are soon raised above their habitual standard; the skin becomes burning; it is dried by an arid heat or covered with an abundant sweat, according as the organs of digestion or of respiration are especially affected."—"The sympathies of the mucous membranes," he adds, "are more pronounced with the portion of the skin which corresponds with them, than with the other parts of the surface of the body. We observe that the heat is greater in the throat, the neck, the thorax, the epigastrium, the umbilical organ, the hypogastrium, according as the membranes which line the pharynx, the larynx, the trachea, the bronchiæ, the stomach, the intestines, the bladder, and the uterus, are the exclusive seat of irritation." These extracts develop the principal points of some views that were more fully exposed in the Exposition of the Doctrine of Broussais, inserted in some late Numbers of this Journal, and in the first of the Gulstonian Lectures of Dr. Harrison. The rest of this section is occupied with some considerations on the sympathies of the mucous membranes with the nervous system; but nothing is adduced that had not been noticed under the heads "Physiology" in the Historical Sketches of the Progress of Medicine, lately inserted in this Journal.

The fourth chapter treats on *diseases in general;* but we have arrived so near the limits of the present Number, that we must defer an abstract of it till the next, when we shall complete the analysis of this work.

---

